# Osteoblast lineage *Sod2* deficiency leads to an osteoporosis-like phenotype in mice

**DOI:** 10.1242/dmm.049392

**Published:** 2022-05-13

**Authors:** Astrid M. Schoppa, Xiangxu Chen, Jan-Moritz Ramge, Anna Vikman, Verena Fischer, Melanie Haffner-Luntzer, Jana Riegger, Jan Tuckermann, Karin Scharffetter-Kochanek, Anita Ignatius

**Affiliations:** 1 Institute of Orthopedic Research and Biomechanics, Ulm University Medical Center, 89081 Ulm, Germany; 2 Department of Orthopedics, Division for Biochemistry of Joint and Connective Tissue Diseases, Ulm University Medical Center, 89081 Ulm, Germany; 3 Institute of Comparative Molecular Endocrinology, Ulm University, 89081 Ulm, Germany; 4 Department of Dermatology and Allergic Diseases, Ulm University Medical Center, 89081 Ulm, Germany

**Keywords:** Skeletal aging, Osteoporosis, Mitochondrial dysfunction, Reactive oxygen species, Senescence

## Abstract

Osteoporosis is a systemic metabolic skeletal disease characterized by low bone mass and strength associated with fragility fractures. Oxidative stress, which results from elevated intracellular reactive oxygen species (ROS) and arises in the aging organism, is considered one of the critical factors contributing to osteoporosis. Mitochondrial (mt)ROS, as the superoxide anion (O_2_^−^) generated during mitochondrial respiration, are eliminated in the young organism by antioxidant defense mechanisms, including superoxide dismutase 2 (SOD2), the expression and activity of which are decreased in aging mesenchymal progenitor cells, accompanied by increased mtROS production. Using a mouse model of osteoblast lineage cells with *Sod2* deficiency, we observed significant bone loss in trabecular and cortical bones accompanied by decreased osteoblast activity, increased adipocyte accumulation in the bone marrow and augmented osteoclast activity, suggestive of altered mesenchymal progenitor cell differentiation and osteoclastogenesis. Furthermore, osteoblast senescence was increased. To date, there are only a few studies suggesting a causal association between mtROS and cellular senescence in tissue *in vivo*. Targeting SOD2 to improve redox homeostasis could represent a potential therapeutic strategy for maintaining bone health during aging.

## Abstract (precis)

**Summary:** Osteoblast-lineage specific *Sod2* deficiency in mice leads to increased mtROS, impaired osteoblast function, increased adipogenesis, increased osteoclast activity and increased osteoblast senescence, resulting in bone loss.

## INTRODUCTION

Osteoporosis is a generalized metabolic skeletal disease, which is characterized by low bone mineral density (BMD) and structural degeneration of the bone tissue, predisposing to high fracture risk, and is one of the major health problems affecting the aging society (Hendrickx et al., 2015). Epidemiological and preclinical studies indicate that reactive oxygen species (ROS) are involved in the development of age-related and postmenopausal osteoporosis (Almeida and O'Brien, 2013; Bonaccorsi et al., 2018; Manolagas, 2010). The ROS level in bone increases with age as the activity of antioxidant defense mechanisms decreases (Almeida et al., 2007; Chung et al., 2011). ROS are mainly generated in the mitochondria during cellular respiration and are eliminated in the young organism by antioxidant defense systems, including the enzyme superoxide dismutase (SOD) 2 (Agidigbi and Kim, 2019; Almeida and O'Brien, 2013). Under physiological conditions, ROS are involved in the regulation of bone remodeling where they facilitate the resorption of bone tissue (Agidigbi and Kim, 2019; Wauquier et al., 2009). They promote bone turnover by increasing osteoclast differentiation and activity in the healthy young organism, whereas in the old organism, increased ROS production can induce the generation of proinflammatory mediators, which both enhance osteoclastic bone degradation and inhibit osteoblastic bone formation (Agidigbi and Kim, 2019).

SODs are the first line of antioxidant defense enzymes against ROS (Younus, 2018). They catalyze the dismutation of the superoxide anion free radical (O_2_^−^) into molecular oxygen and hydrogen peroxide; the latter is converted by catalase to water. SODs serve as anti-inflammatory agents and SOD conjugates have been demonstrated as potential therapeutic agents in age-related and inflammatory diseases, including neutrophil-mediated inflammation (Fang et al., 2009; Younus, 2018). In humans, three forms of SODs are present: the Cu- and Zn-containing SOD1 and SOD3, which are located in the cytoplasm and the extracellular compartment, respectively, and the Mn-containing SOD2, which is located in the mitochondria and is necessary for eliminating superoxide radicals released mainly from the mitochondrial complex III during cellular respiration, the main source of ROS within a cell (Bigarella et al., 2014). During osteoblast differentiation, SOD2 is upregulated to maintain mitochondrial function and osteoblast differentiation (Gao et al., 2018).

It has been shown that rat and human mesenchymal progenitor cell aging is associated with decreased SOD2 expression and activity and increased mitochondrial ROS (mtROS) production (Almeida and O'Brien, 2013; Chen et al., 2019; Stolzing et al., 2008; Stolzing and Scutt, 2006). Decreased SOD2 activity and mitochondrial oxidative stress have been demonstrated to be associated with senescence in the skin and brain (Flynn and Melov, 2013; Melov et al., 1999; Velarde et al., 2012). Cellular senescence is considered as a stress response normally induced by various extrinsic and intrinsic insults, including irradiation, oxidative stress and mitochondrial dysfunction, and was originally identified as a highly stable cell cycle arrest (Qadir et al., 2020). Recent studies indicated a crucial role of mitochondrial oxidative stress and functional SOD2 in implant osteointegration (Wang et al., 2020; Zhou et al., 2019). How mitochondrial oxidative damage affects bone integrity due to reduced SOD2 activity in osteoblast lineage cells is still unknown. Therefore, we established and characterized a mouse model of *Sod2* deficiency in the osteoblast lineage to study the influence of ROS specifically generated in osteoblast lineage cells on bone metabolism. We found that the increased mtROS generation in osteoblast lineage cells resulted in an accumulation of adipocytes in the bone marrow, altered osteoblast and osteoclast activity as well as increased osteoblast senescence and bone loss *in vivo*, implying SOD2 as a potential target for maintaining bone health during aging.

## RESULTS

### Osteoblast lineage-specific *Sod2* deficiency leads to bone loss in mice

**Fig. 1.**
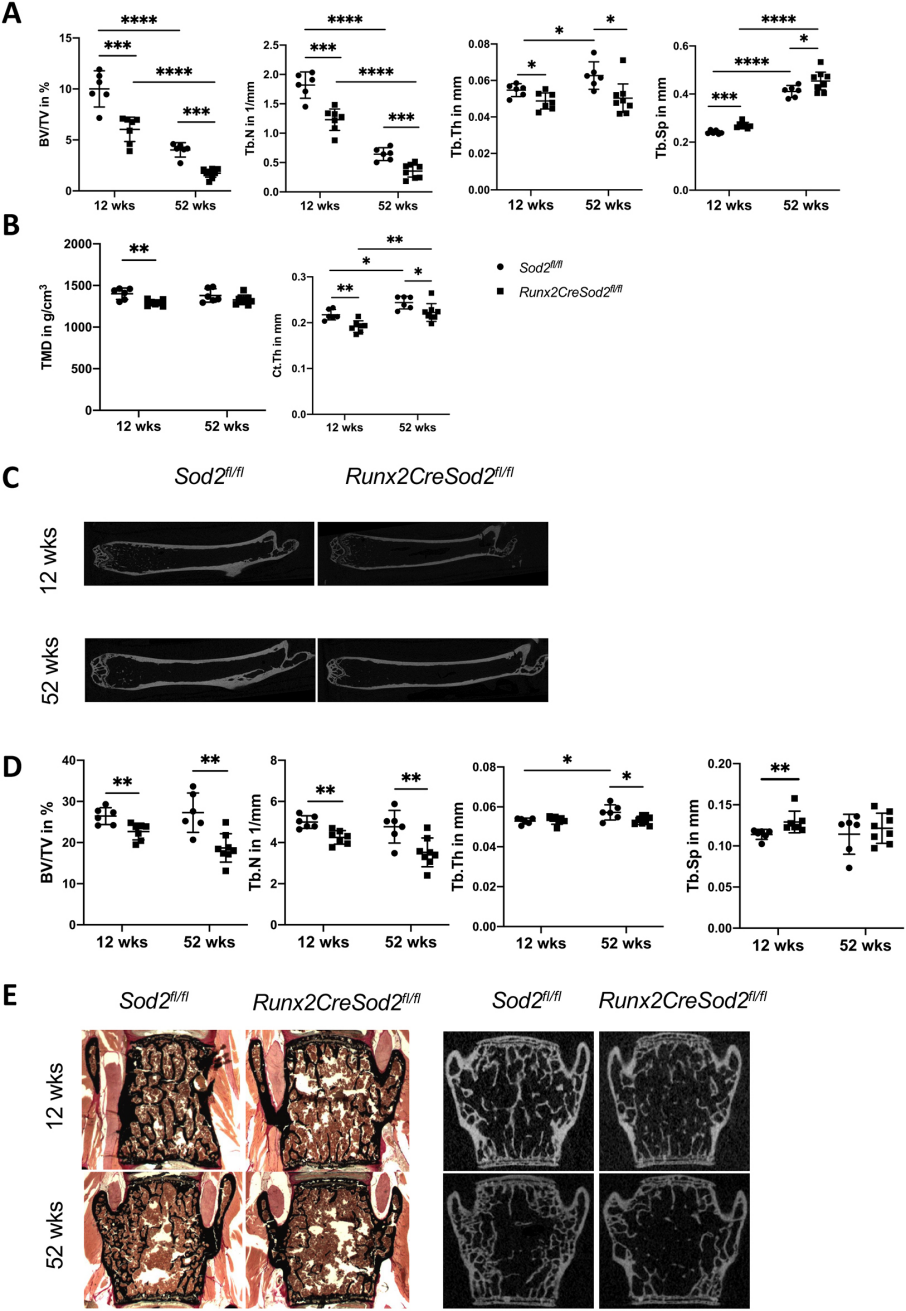
**Bone mass loss in osteoblast lineage-specific *Sod2*-deficient female mice aged 12 and 52 weeks.** (A) µCT-based quantification of the trabecular bone volume per tissue volume ratio (BV/TV), trabecular number (Tb.N), trabecular thickness (Tb.Th) and trabecular separation (Tb.Sp) in the distal femora of *Runx2CreSod2^fl/fl^* mice (◼) compared to *Sod2^fl/fl^* mice (•). (B) µCT-based quantification of the TMD and cortical thickness (C.Th) of the femora of *Runx2CreSod2^fl/fl^* mice (◼) compared to *Sod2^fl/fl^* mice (•). (C) Representative two-dimensional (2D) µCT images of the femora. (D) Histomorphometric quantification of BV/TV, Tb.N, Tb.Th and Tb.Sp in the vertebral bodies of *Runx2CreSod2^fl/fl^* mice (◼) compared to *Sod2^fl/fl^* mice (•) determined by µCT. (E) Representative histological images of vertebral body sections after Von Kossa staining and representative 2D µCT images of vertebral bodies. Bars represent mean±s.d. (*n*=6-7/group). Asterisks indicate statistically significant differences between the groups (*P<0.05, **P<0.01, ***P<0.001, ****P<0.001; two-way ANOVA).

To analyze the effect of osteoblast lineage-specific *Sod2* deficiency on bone mass in mice, we deleted *Sod2* by crossing *Sod2^fl/fl^* mice with *Runx2Cre* mice, which express Cre recombinase in osteoblast lineage cells (Rauch et al., 2010; Strassburger et al., 2005; Treiber et al., 2011). Female mice lacking *Sod2* expression, hereafter referred to as *Runx2CreSod2^fl/fl^*, displayed a decreased trabecular bone volume per tissue volume ratio (BV/TV), trabecular number (Tb.N), trabecular thickness (Tb.Th) and an increased trabecular separation (Tb.Sp) at 12 and 52 weeks of age, as determined by micro-computed tomography (µCT) in the femora, compared to their littermate control mice (Fig. 1A). *Runx2CreSod2^fl/fl^* mice aged 12 weeks revealed a bone volume fraction that was not significantly different from *Sod2^fl/fl^* mice at the age of 52 weeks. Moreover, female *Runx2CreSod2^fl/fl^* mice displayed decreased tissue mineral density (TMD) and cortical thickness (Ct.Th) compared to control mice (Fig. 1B,C). In the vertebrae of female *Runx2CreSod2^fl/fl^* mice, the trabecular bone volume fraction and Tb.N were decreased and Tb.Sp was increased (Fig. 1D,E). Male mice with osteoblast lineage-specific *Sod2* deficiency also displayed a decreased BV/TV, Tb.N and Tb.Th and an increased Tb.Sp at 12 and 52 weeks of age, as determined by µCT in the femora, compared to their littermate control mice (Fig. S1A). In male *Runx2CreSod2^fl/fl^* mice aged 12 and 52 weeks, cortical TMD and Ct.Th were also decreased (Fig. S1B).

Efficient recombination by *Runx2Cre* was also previously observed at sites of endochondral bone formation (Rauch et al., 2010), and thus we analyzed the growth plate thickness and found a significantly decreased growth plate thickness in the femora of female *Runx2CreSod2^fl/fl^* mice aged 12 weeks (Fig. S2), whereas male mice did not show significant growth plate thickness changes (results not shown). Because osteoblast lineage-specific *Sod2* deficiency in female mice led to a more pronounced femoral trabecular skeletal phenotype compared to male mice, we used the long bones of female mice for further analyses, unless otherwise specified.

**Fig. 2.**
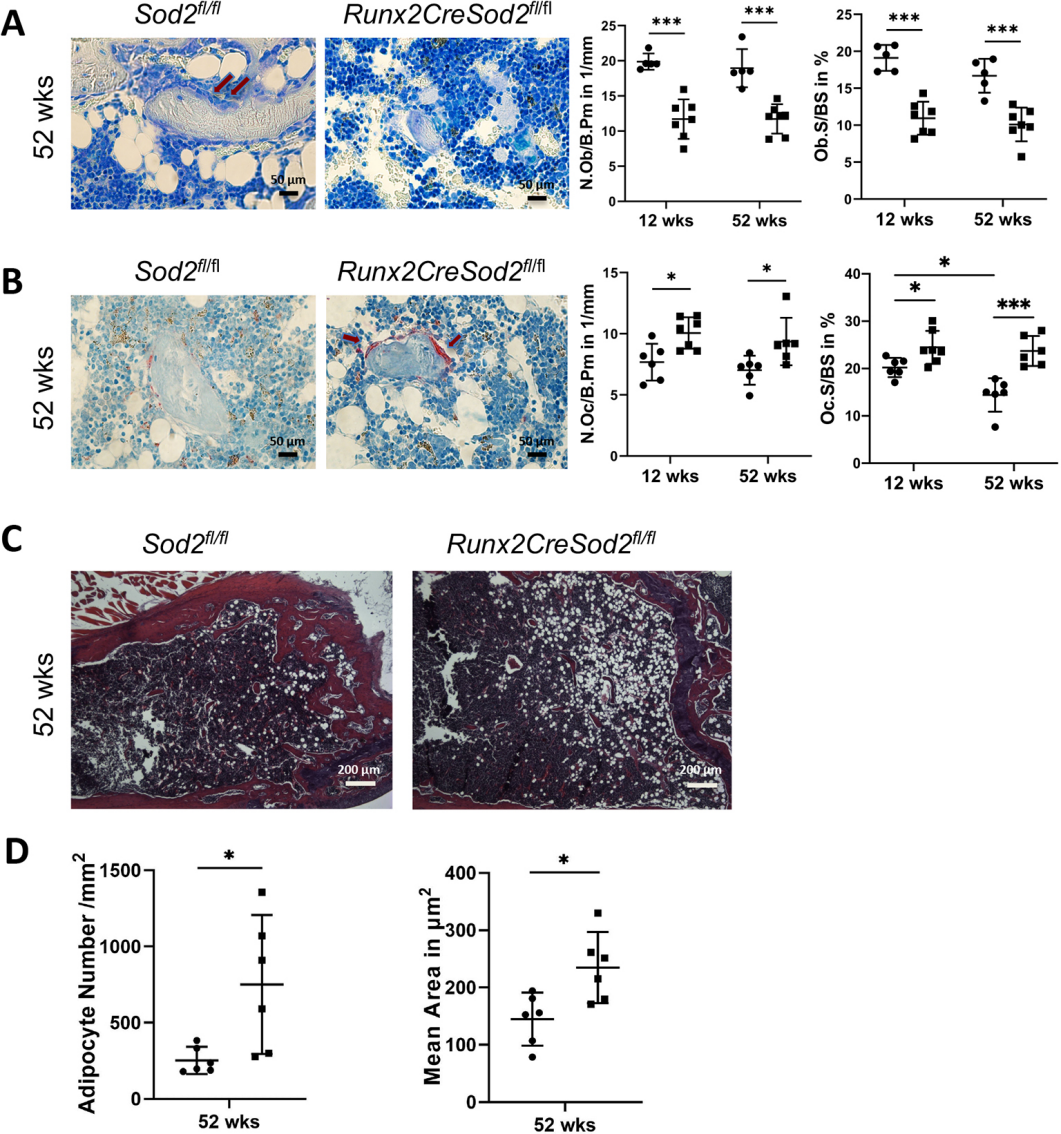
**Bone mass loss is caused by a decreased number of osteoblasts and an increased number of osteoclasts in osteoblast lineage-specific *Sod2*-deficient mice aged 12 and 52 weeks.** (A) Representative images of femur cross-sections stained with Toluidine Blue from *Sod2^fl/fl^* mice and *Runx2CreSod2^fl/fl^* mice. Histomorphometric analysis of osteoblast number per bone perimeter (N.Ob/B.Pm) and osteoblast surface per bone surface (Ob.S/BS) in the femora of *Runx2CreSod2^fl/fl^* mice (◼) compared to *Sod2^fl/fl^* mice (•). Red arrows indicate osteoblasts. Scale bars: 50 μm. (B) Representative images of femur cross-sections stained for tartrate-resistant acid phosphatase (TRAP) from *Sod2^fl/fl^* mice and *Runx2CreSod2^fl/fl^* mice. Histomorphometric analysis of osteoclast number per bone perimeter (N.Oc/B.Pm) and osteoclast surface per bone surface (Oc.S/BS) in the femora of *Runx2CreSod2^fl/fl^* mice (◼) compared to *Sod2^fl/fl^* mice (•). Red arrows indicate osteoclasts. Scale bars: 50 μm. (C) Representative images of femur cross-sections stained with Hematoxylin and Eosin (HE). Scale bars: 200 μm. (D) Quantification of adipocytes identified as ghost-like cells. Bars represent mean±s.d. (*n*=6-7/group). Asterisks indicate statistically significant differences between the two groups (*P<0.05, ***P<0.001; two-way ANOVA and two-tailed unpaired *t*-test).

To analyze the cellular changes underlying the skeletal phenotype of female *Runx2CreSod2^fl/fl^* mice, bone histomorphometry was performed and revealed a lower osteoblast number (Fig. 2A) and an increased osteoclast number (Fig. 2B) in *Runx2CreSod2^fl/fl^* mice when aged 12 and 52 weeks. The cortical bone marrow area displayed a higher adipocyte number and adipocyte area in the femora of female *Runx2CreSod2^fl/fl^* mice aged 52 weeks (Fig. 2C,D).

### Higher ROS generation in *Sod2*-deficient osteoblast lineage cells impairs the proliferation and differentiation of osteoblasts and mesenchymal osteoprogenitor cells

**Fig. 3.**
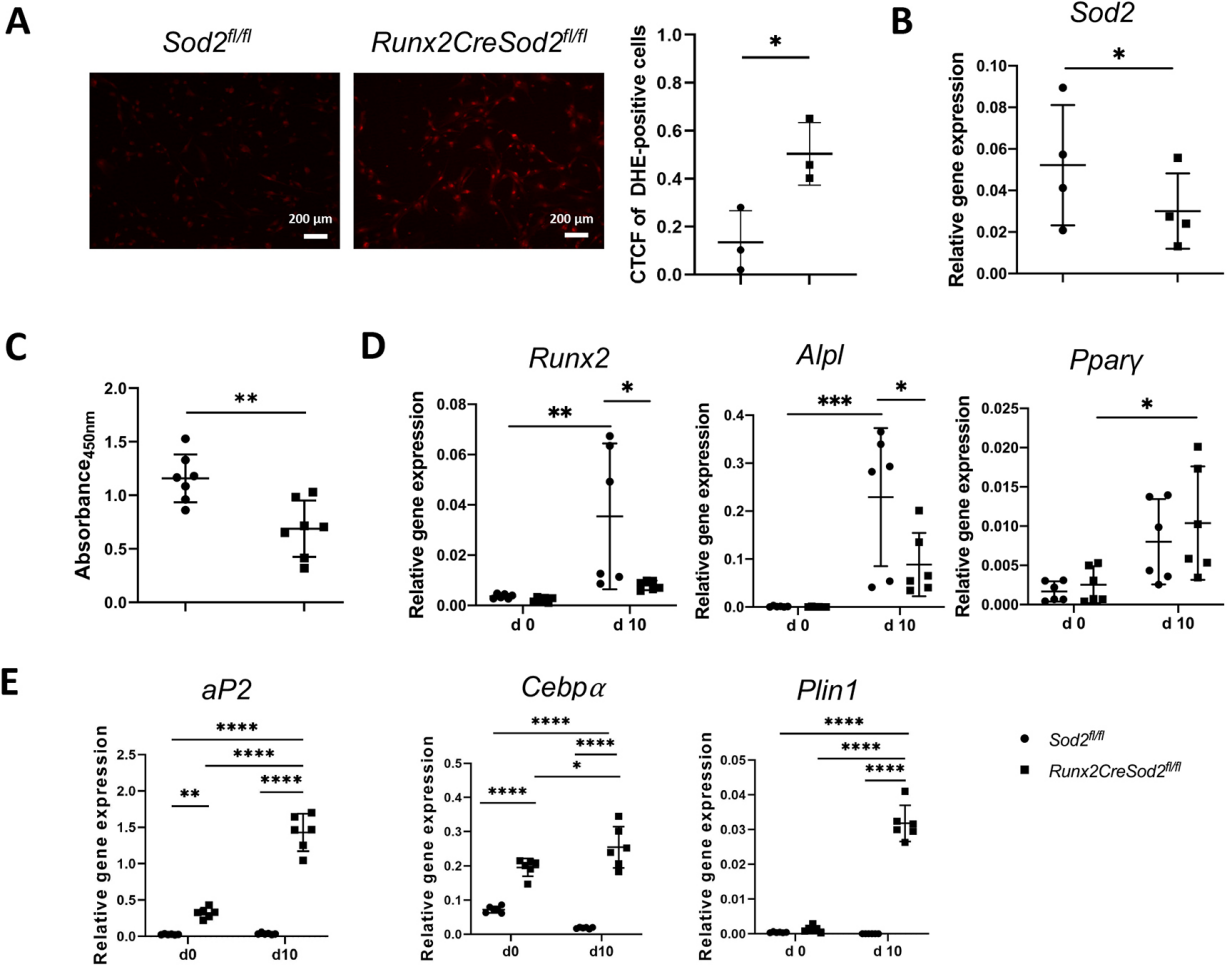
***Sod2* deficiency in the osteoblast lineage leads to higher ROS generation and impaired osteoblast proliferation and impaired mesenchymal osteoprogenitor function with increased adipogenic marker expression.** (A) Detection of ROS generation in osteoblasts from *Sod2^fl/fl^* mice and *Runx2CreSod2^fl/fl^* mice *in vitro* by the dihydroethidium (DHE) assay and quantification by calculating the corrected total cell fluorescence (CTCF) of DHE-positive cells. Scale bars: 200 μm. (B) qRT-PCR analysis of *Sod2* expression in osteoblasts from *Sod2^fl/fl^* mice (•) and *Runx2CreSod2^fl/fl^* mice (◼). (C) Quantification of osteoblast proliferation from *Sod2^fl/fl^* mice (•) and *Runx2CreSod2^fl/fl^* mice (◼) by BrdU assay. (D) qRT-PCR analysis of *Runx2*, *Alpl* and *Pparg* expression in mesenchymal osteoprogenitor cells from *Sod2^fl/fl^* mice (•) and *Runx2CreSod2^fl/fl^* mice (◼) on day 0 (d 0, third day after seeding) and additionally after 10 days (d 10) of cultivation in differentiation medium. (E) qRT-PCR analysis of *aP2, Cebpa* and *Plin1* expression in mesenchymal osteoprogenitor cells from *Sod2^fl/fl^* mice (•) and *Runx2CreSod2^fl/fl^* mice (◼) on day 0 (third day after seeding) and additionally after 10 days (day 10) of cultivation in osteogenic differentiation medium. Bars represent mean±s.d. (*n*=3-7/group). Asterisks indicate statistically significant differences between the groups (*P<0.05, **P<0.01, ***P<0.001, ****P<0.0001; two-way ANOVA and two-tailed unpaired *t*-test).

**Fig. 4.**
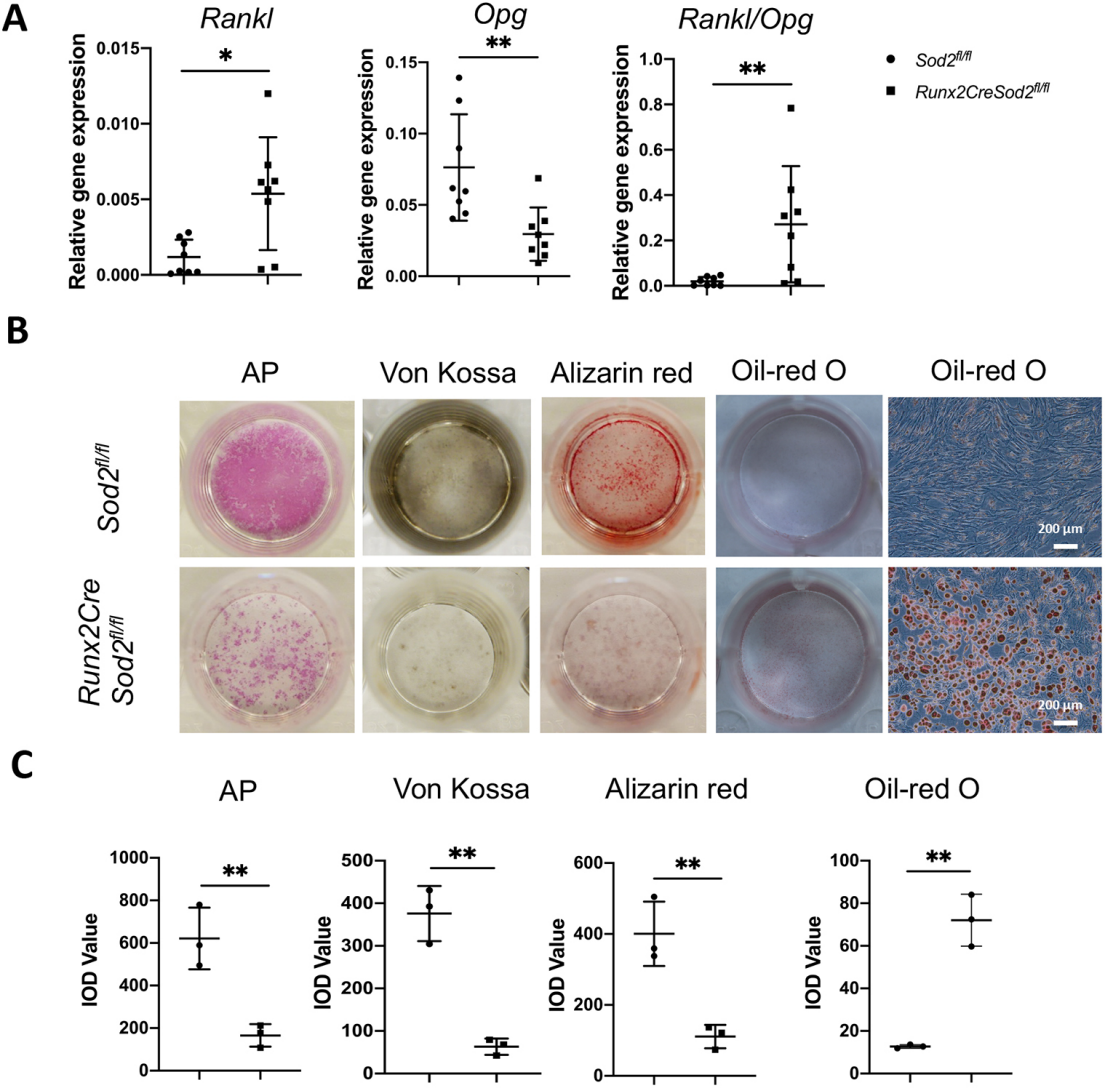
***Sod2* deficiency in the osteoblast lineage leads to impaired osteoblast function and mesenchymal osteoprogenitor differentiation.** (A) qRT-PCR analysis of *Rankl* and *Opg* expression and *Rankl/Opg* ratio in osteoblasts from *Sod2^fl/fl^* mice and *Runx2CreSod2^fl/fl^* mice after cultivation in osteogenic differentiation medium for 14 days. (B) Representative images of osteoblast cultures from *Sod2^fl/fl^* mice and *Runx2CreSod2^fl/fl^* mice on day 14 of cultivation in osteogenic differentiation medium and alkaline phosphatase (AP), Von Kossa and Alizarin Red staining, and representative images of mesenchymal osteoprogenitor cell cultures from *Sod2^fl/fl^* mice and *Runx2CreSod2^fl/fl^* mice after Oil Red O staining after cultivation in adipogenic differentiation medium for 3 days. Scale bars: 200 µm. (C) Quantification by calculating the integrated optical density (IOD). Bars represent mean±s.d. (*n*=3-8/group). Asterisks indicate statistically significant differences between the two groups (*P<0.05, **P<0.01; two-tailed unpaired *t*-test).

Dihydroethidium (DHE) staining of osteoblasts in culture was performed to analyze ROS generation due to osteoblast lineage-specific *Sod2* deficiency. Osteoblasts from *Runx2CreSod2^fl/fl^* mice revealed increased ROS generation by an increased corrected total cell fluorescence (CTCF) of DHE-positive cells compared to osteoblasts from control mice (Fig. 3A). We detected significantly reduced *Sod2* expression in osteoblasts isolated from the long bones of *Runx2CreSod2^fl/fl^* mice in comparison with its expression in osteoblasts from *Sod2^fl/fl^* mice (Fig. 3B). Proliferation of osteoblasts of *Sod2^fl/fl^* and *Runx2CreSod2^fl/fl^* mice was analyzed by bromodeoxyuridine (BrdU) assay. The proliferation of osteoblasts from *Runx2CreSod2^fl/fl^* mice was significantly reduced (Fig. 3C). To investigate the influence of higher ROS generation in osteoblast lineage cells on osteogenic differentiation, both mesenchymal osteoprogenitor cells and osteoblasts from *Sod2^fl/fl^* and *Runx2CreSod2^fl/fl^* mice were cultured in differentiation medium. Mesenchymal osteoprogenitor cells were cultured for 10 days and osteoblasts were cultured for 14 days in osteogenic differentiation medium. As expected, the expression of the osteogenic differentiation markers runt-related transcription factor 2 (*Runx2*) and alkaline phosphatase (*Alpl*) was increased at day 10 of differentiation in osteoprogenitor cells from *Sod2^fl/fl^* mice (Fig. 3D). *Runx2* and *Alpl* expression was decreased at day 10 in osteoprogenitor cells from *Runx2CreSod2^fl/fl^* mice compared to their expression in osteoprogenitor cells from *Sod2^fl/fl^* mice. At day 10, peroxisome proliferator-activated receptor gamma (*Pparg*) expression was upregulated in osteoprogenitor cells from *Sod2^fl/fl^* mice, whereas the upregulation in osteoprogenitor cells from *Runx2CreSod2^fl/fl^* mice tended to be higher. Moreover, the adipogenesis markers CCAAT/enhancer binding protein alpha (*Cebpa*), adipocyte P2 (*aP2*, also known as *Fabp4*) and perilipin1 (*Plin1*) were significantly upregulated in osteoprogenitor cells with *Sod2* deficiency (Fig. 3E). In osteoblasts from *Runx2CreSod2^fl/fl^* mice, the ratio of receptor activator of nuclear factor kappa B ligand (*Rankl*) to osteoprotegerin (*Opg*) was significantly increased compared to osteoblasts from control mice (Fig. 4A). In agreement with the decreased *Runx2* and *Alpl* expression, osteoblast differentiation was impaired, as revealed by reduced alkaline phosphatase (AP) staining as well as reduced Von Kossa and Alizarin Red staining in osteoblast cultures of *Runx2CreSod2^fl/fl^* mice (Fig. 4B,C). Oil Red O staining demonstrated higher adipogenesis in mesenchymal osteoprogenitor cultures from *Runx2CreSod2^fl/fl^* mice (Fig. 4B,C).

### Increased senescence of osteoblasts in osteoblast lineage-specific *Sod2*-deficient mice

**Fig. 5.**
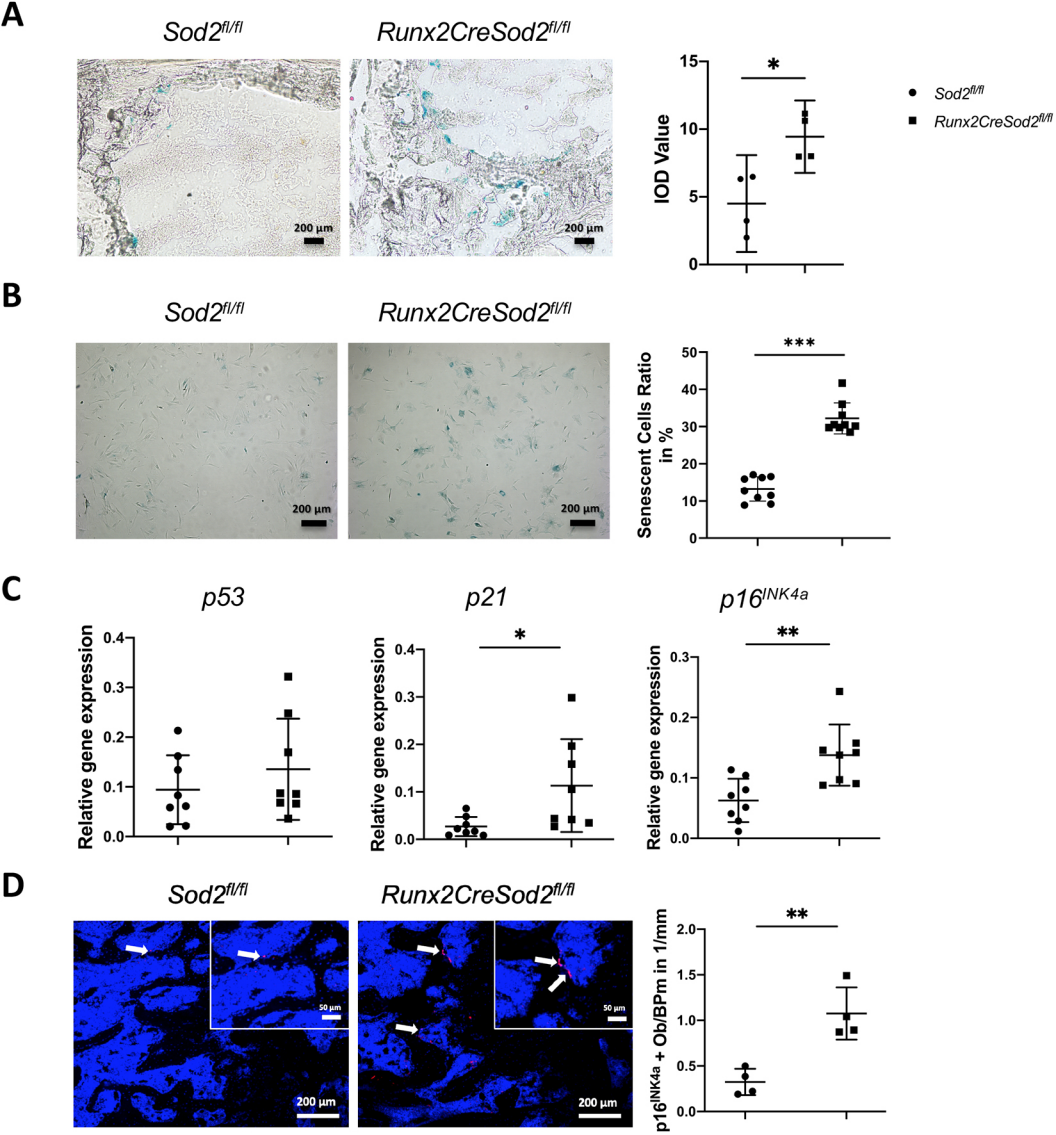
***Sod2* deficiency in osteoblast lineage cells leads to senescence *in vitro* and *in vivo*.** (A) Representative images of senescent osteoblasts from *Sod2^fl/fl^* mice and *Runx2CreSod2^fl/fl^* mice detected *in situ* (tibia cryosections) by histochemical staining of senescence-associated β-galactosidase (SA-β-Gal) activity (blue) and quantification by calculating the IOD. (B) Detection and quantification of senescent osteoblasts from *Sod2^fl/fl^* mice (•) and *Runx2CreSod2^fl/fl^* mice (◼) *in vitro* based on cytochemical staining of SA-β-Gal activity (blue). (C) qRT-PCR analysis of senescence-associated tumor suppressor markers *p53*, *p21* and *p16^INK4a^* expression in osteoblasts from *Sod2^fl/fl^* mice (•) and *Runx2CreSod2^fl/fl^* mice (◼) *in vitro*. (D) Representative immunofluorescence images of femur cross-sections stained for p16^INK4a^ (arrows indicate p16^INK4a^-positive cells) and quantification of the number of p16^INK4a^-positive cells in femur cross-sections from *Sod2^fl/fl^* mice (•) and *Runx2CreSod2^fl/fl^* mice (◼). Bars represent mean±s.d. (*n*=4-9/group). Asterisks indicate statistically significant differences between the two groups (*P<0.05, **P<0.01, ***P<0.001; two-tailed unpaired *t*-test). Scale bars: 200 μm and 50 μm (insets).

We applied the senescence-associated β-galactosidase (SA-β-Gal) activity assay to analyze the influence of osteoblast lineage-specific *Sod2* deficiency on the senescence status of osteoblasts *in vivo*. Obvious blue staining indicating SA-β-Gal activity in osteoblasts on the surface of bone trabeculae in the femora in both *Sod2^fl/fl^* and *Runx2CreSod2^fl/fl^* mice aged 52 weeks was observed, although osteoblast-specific *Sod2* deficiency led to an increased osteoblastic SA-β-Gal activity staining (Fig. 5A). To verify the increased SA-β-Gal activity observed *in vivo*, we stained osteoblasts in cultures for SA-β-Gal activity. The number of osteoblasts with SA-β-Gal activity from *Runx2CreSod^fl/fl^* mice in culture was increased compared to that of SA-β-Gal activity-positive osteoblasts from *Sod2^fl/fl^* mice (Fig. 5B).

Furthermore, we analyzed senescence-associated gene marker expression. The expression of the tumor suppression markers *p21* and *p16^INK4a^* was significantly increased in osteoblasts from *Runx2CreSod2^fl/fl^* mice (Fig. 5C). Consistent with this, we detected more *p16^INK4a^*-positive osteoblasts on the trabecular surfaces in the femora of *Runx2CreSod2^fl/fl^* mice (Fig. 5D).

**Fig. 6.**
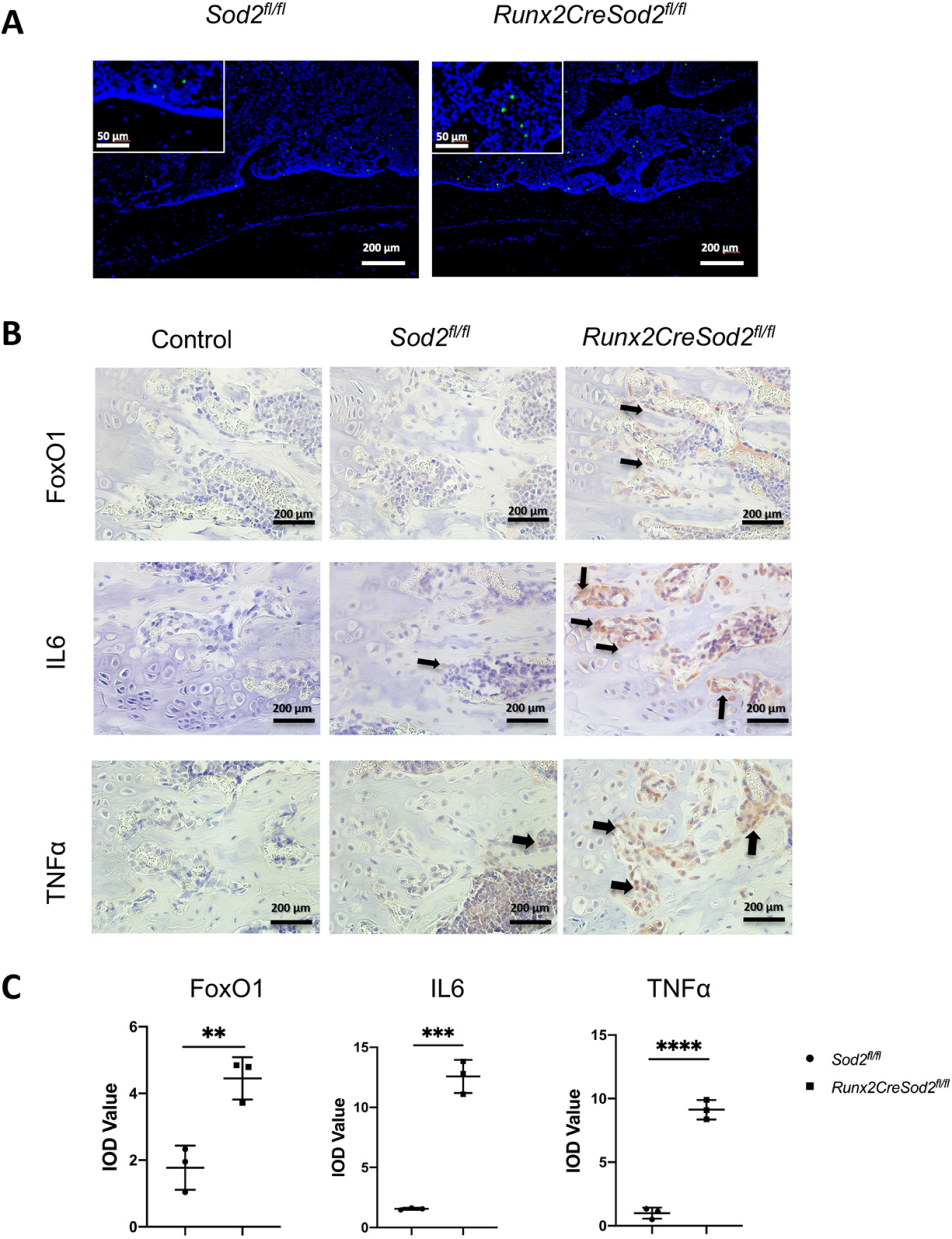
***Sod2* deficiency in osteoblast lineage cells does not increase osteoblast apoptosis but leads to increased expression of senescence-associated secretory phenotype (SASP) markers and FoxO1.** (A) Representative images of femur cross-sections from *Sod2^fl/fl^* mice and *Runx2CreSod2^fl/fl^* mice after TUNEL assay to detect apoptosis. (B) Representative images of femur cross-sections from male *Sod2^fl/fl^* mice and *Runx2CreSod2^fl/fl^* mice after immunostaining for the SASP factors IL-6 and TNF-α and the redox-regulated transcription FoxO1. Arrows indicate positive staining for FoxO1, IL-6 and TNF-α expression in osteoblast lineage cells. (C) Quantification by calculating the IOD. Bars represent mean±s.d. (*n*=3/group). Asterisks indicate statistically significant differences between the groups (**P<0.01, ***P<0.001, ****P<0.0001; two-tailed unpaired *t*-test). Scale bars: 200 μm and 50 μm (insets).

**Fig. 7.**
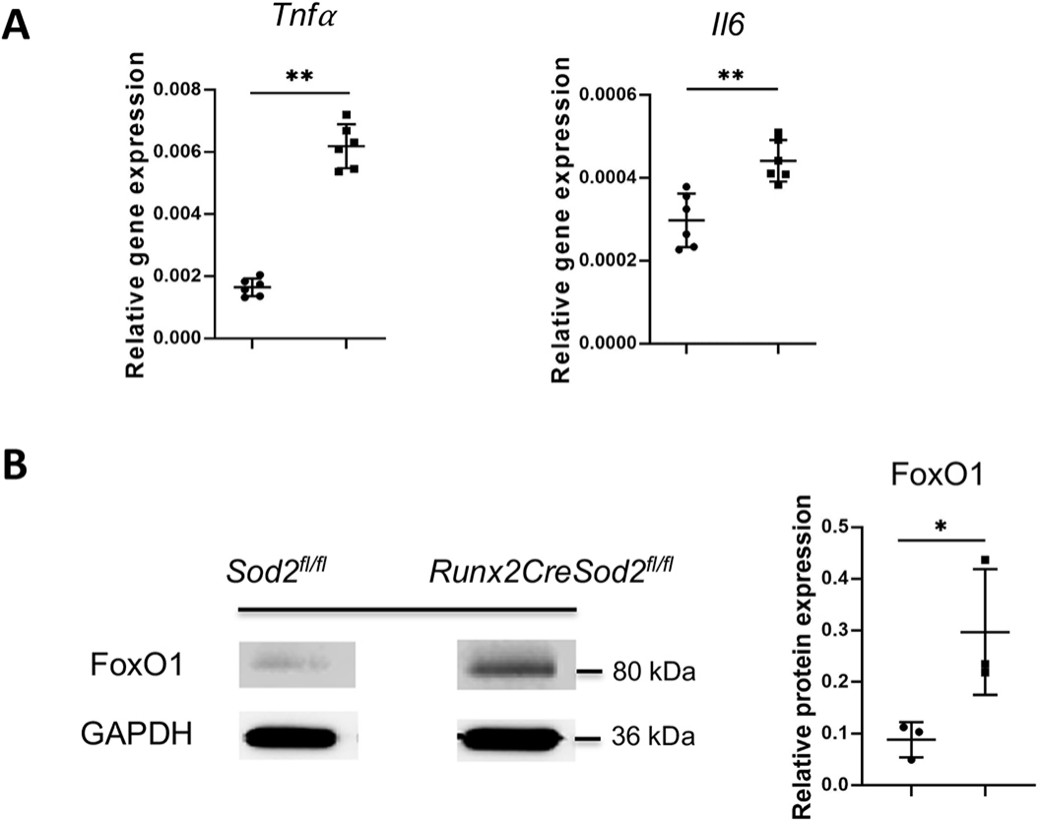
**Increased expression of senescence-associated secretory-phenotype (SASP) markers *Tnfa*, *Il6* and FoxO1 in mesenchymal osteoprogenitor cells with *Sod2* deficiency.** (A) qRT-PCR analysis of senescence-associated markers *Tnfα* and *Il6* expression in mesenchymal osteoprogenitor cells after osteogenic differentiation from *Sod2^fl/fl^* mice (•) and *Runx2CreSod2^fl/fl^* mice (◼) *in vitro*. (B) Western blot analysis of FoxO1 expression in osteoblasts from *Sod2^fl/fl^* mice (•) and *Runx2CreSod2^fl/fl^* mice (◼) and quantification by calculating the IOD. Bars represent mean±s.d. (*n*=6/group). Asterisks indicate statistically significant differences between the groups (*P<0.05, **P<0.01; two-tailed unpaired *t*-test).

We could not detect any differences in apoptotic osteoblasts in the bone tissue of the two mouse genotypes (Fig. 6A). Moreover, we analyzed the senescence-associated secretory phenotype (SASP) marker expression causing premature senescence (Qadir et al., 2020) in osteoblasts. SASP factors interleukin 6 (IL-6) and tumor necrosis factor α (TNF-α, also known as TNF) were upregulated in osteoblasts surrounding the trabecular bone in the femora of *Runx2CreSod2^fl/fl^* mice in comparison with *Sod2^fl/fl^* mice (Fig. 6B,C). The expression of the transcription factor FoxO1, which preserves redox balance to ensure bone redox homeostasis (Ma et al., 2020), was also upregulated in response to ROS in osteoblasts enclosing the trabecular bone of *Runx2CreSod2^fl/fl^* mice compared to *Sod2^fl/fl^* mice (Fig. 6B,C). Real-time quantitative reverse transcription PCR (qRT-PCR) expression analysis confirmed the upregulation of *Tnfa* and *Il6* in mesenchymal osteoprogenitor cells from *Runx2CreSod2^fl/fl^* mice (Fig. 7A). Accordingly, FoxO1 protein expression was also upregulated in isolated osteoblasts from *Runx2CreSod2^fl/fl^* mice, as shown by western blotting (Fig. 7B).

## DISCUSSION

Although it has been reported that oxidative stress in osteoblasts plays a significant role in the pathogenesis of osteoporosis (Almeida et al., 2007; Rached et al., 2010), with SOD2 being important for removal of excessive ROS (Younus, 2018), the exact molecular and functional consequences of mtROS accumulation in osteoblastic cells are still not completely understood. To our best knowledge, there is only one preclinical study in bone cell-specific *Sod2*-deficient mice (*Dmp1CreSod2^fl/fl^*), which allows the investigation of oxidative stress selectively in late osteoblasts and terminally differentiated osteocytes (Kobayashi et al., 2015), but not in differentiating osteoblast precursor cells. Although decreased SOD2 activity and subsequent disbalance in mtROS have previously been described in aging mesenchymal progenitor cells (Almeida and O'Brien, 2013; Chen et al., 2019; Stolzing et al., 2008; Stolzing and Scutt, 2006), the consequences on bone integrity and development of osteoporosis have not been addressed before. Using our new mouse model, which is characterized by an osteoblast lineage-specific *Sod2* knockout, we provide a novel insight into the consequences of *Sod2* deficiency along the osteoblast lineage including progenitor cells. Overall, we demonstrated that mtROS accumulation in early osteoblast precursor cells considerably disturbed osteogenic differentiation with a switch to the adipogenic lineage, induced osteoblast senescence and promoted osteoclast formation, resulting in remarkable bone loss. These results are meaningful for a better understanding of aberrant antioxidative defense mechanisms in osteogenic cells and subsequent development of age-related osteoporosis.

Osteoblast lineage-specific *Sod2* deficiency in female and male mice resulted in a low bone mass phenotype that was characterized by a decreased trabecular bone volume fraction, when aged 12 and 52 weeks in the femora. Interestingly, the decrease in the bone mass of *Runx2CreSod2^fl/fl^* mice at the age of 12 weeks corresponded to that observed in *Sod2^fl/fl^* control mice aged 52 weeks, indicating that *Sod2* deficiency in part resembles an age-related bone phenotype. As in humans, femoral trabecular bone mass decreases with age in mice, starting in early adulthood before any changes in sex steroid production occur (Jilka, 2013). We also found a decreased cortical mineral density and thickness of the femoral bone in *Runx2CreSod2^fl/fl^* mice with *Sod2* deficiency in osteoblast lineage cells. These results are consistent with previous studies in mice with a global knockout of cytoplasmatic *Sod1* (Nojiri et al., 2011). *Sod1*-deficient mice also displayed low bone mass that was accompanied by increased ROS generation in osteoblasts derived from these mice (Nojiri et al., 2011). A further study (Kobayashi et al., 2015) demonstrated that *Sod2* depletion in osteocytes leads to a remarkable bone loss in an age-dependent manner. These and our results confirm other studies demonstrating that oxidative stress is involved in age-related bone loss (Almeida, 2012; Jilka, 2013). Clinical studies showed that elevated oxidative stress is associated with the pathogenesis of osteoporosis (Bonaccorsi et al., 2018; Deveci et al., 2017; Domazetovic et al., 2017). In particular, one clinical study demonstrated that aged postmenopausal women exhibit a significant increase in serum lipid hydroperoxides, a ROS-induced byproduct, accompanied by a decreased BMD in vertebrae, compared to young women in the reproductive age (Cervellati et al., 2014), suggesting that estrogen deficiency promotes oxidative stress, leading to low bone mass.

Overall, it should also be mentioned that global knockout of *Sod2* results in neonatal lethality, whereas *Sod1* deficiency leads to shortened mean lifespan and various age-related pathophysiological changes in different tissues and organs (Watanabe et al., 2014). Although both global knockout of cytoplasmic *Sod1* as well as osteocyte-specific knockout of mitochondrial *Sod2* seem to result in an osteoporosis-like phenotype in mice (Nojiri et al., 2011; Kobayashi et al., 2015), several studies imply differences in the expression and the role of these antioxidative enzymes in bone. While *Sod1* was upregulated in bone marrow cells in response to mechanical unloading-induced ROS generation and seemed to be protective against subsequent bone loss, *Sod2* was not involved in the antioxidative response (Morikawa et al., 2013). However, in contrast to mitochondrial SOD2, which plays a pivotal role in counterbalancing excessive ROS production during osteoblast differentiation and bone formation, expression of *Sod1* was not found to be upregulated during this process (Gao et al., 2018). Considering the various pathological changes observed in *Sod1^−/−^* mice, which might indirectly affect bone physiology, an osteoblast-specific *Sod1* knockout model might help to differentiate the respective roles of SOD1 and SOD2 in bone development and metabolism.

The bone loss in *Runx2CreSod2^fl/fl^* mice might result from an impaired osteoblast and elevated osteoclast activity, because we detected a decreased osteoblast number and osteoblast surface as well as an increased osteoclast number and osteoclast surface in these mice. Furthermore, adipocyte number in the bone marrow of *Sod2*-deficient mice was increased, suggesting altered mesenchymal progenitor cell differentiation.

Consistent with the expectation that *Runx2*-controlled Cre expression might also occur in hypertrophic chondrocytes (Rauch et al., 2010; Elefteriou and Yang, 2011), we found a significantly decreased growth plate thickness in the femora of female *Runx2CreSod2^fl/fl^* mice, indicating a disturbed metabolism in the presence of increased ROS levels in these cells, as shown previously (Bai et al., 2019). Although the underlying mechanisms have not been addressed in the present study, we assume that excessive ROS accumulation associated with endochondral ossification in the growth plate and simultaneous loss of *Sod2* due to *Runx2* expression in hypertrophic chondrocytes might result in increased cellular stress. This interesting finding deserves further investigation to clarify whether excessive oxidative stress results in apoptotic cell death or any other cellular alteration in the growth plate.

Supporting these *in vivo* findings, osteoblasts isolated from *Sod2*-deficient mice also exhibited an increased ROS accumulation, and mesenchymal progenitor cells isolated from these mice exhibited reduced *Runx2* and *Alpl* expression with a concomitant increased *Pparg*, *Cebpa*, *aP2* and *Plin1* expression, suggesting disturbed osteogenic differentiation and a switch to the adipogenic lineage. Moreover, the ratio of *Rankl* to *Opg* expression, which is important for osteoclastogenesis, was elevated in osteoblasts from *Runx2CreSod2^fl/fl^* mice, which explains the higher osteoclast activity observed in these mice. These results are consistent with Kobayashi et al. (2015), who demonstrated significantly suppressed bone formation and increased bone resorption concomitant with an upregulation of the *Rankl*/*Opg* ratio in a mouse model of osteocyte-specific *Sod2* deletion. Furthermore, in our study, a lower BrdU incorporation and a decreased AP, Von Kossa and Alizarin Red staining of differentiated osteoblast cultures, as well as a higher adipocyte number in mesenchymal progenitor cell cultures originated from *Runx2CreSod2^fl/fl^* mice, also indicated an impaired proliferation and osteogenic differentiation potential. Thus, osteoblast lineage-specific *Sod2* deficiency resulted in bone loss, which was closely associated with impaired osteoblast differentiation and bone marrow adipogenesis, as well as with increased osteoclastogenesis.

Of note, the composition of the bone marrow also changed with aging in humans, showing a high accumulation of adipocytes (Gao et al., 2014; Sebo et al., 2019). The sites of accumulated adipocytes in the bone marrow are referred to as marrow adipose tissue (MAT). MAT is an important endocrine organ, which is able to regulate the systemic metabolism. Interestingly, it was demonstrated to be inversely related to BMD in humans. Moreover, MAT is also associated with metabolic diseases, including metabolic syndrome and diabetes mellitus, and plays a crucial role in the development and progression of tumors, including bone metastasis.

Notably, we discovered more senescent osteoblasts both in osteoblast cultures and in the bone tissue of *Runx2CreSod2^fl/fl^* mice by detecting SA-β-Gal activity. Common characteristics of senescent cells are irreversible growth arrest, the development of a unique secretome, known as the SASP, and the resistance to apoptosis (Farr and Almeida, 2018). Consistent with the importance of the p53/p21 and p16/Rb tumor-suppressor pathways in inducing cellular senescence (Moussavi-Harami et al., 2004), we detected a significant increase of *p21* and *p16^INK4a^* expression in *Sod2-*deficient osteoblasts, suggesting that ROS induced growth arrest of these cells is dependent on the activation of both these tumor-suppressor pathways. Accordingly, we also detected more *p16^INK4a^*-positive osteoblasts *in situ*. Despite the increased oxidative stress level in the bone tissue of *Runx2CreSod2^fl/fl^* mice, apoptosis was not increased in osteoblast lineage cells in these mice, as could be expected due to the resistance of senescent cells to apoptosis (Farr and Almeida, 2018).

Chronic inflammation, which is associated with aging and thus also referred to as ‘inflamm-aging’ (Xia et al., 2016), plays a crucial role in age-related bone loss through the actions of proinflammatory cytokines (Pietschmann et al., 2016). Elevated oxidative stress, particularly mtROS, is thought to be involved in inducing inflammatory cytokines via activation of nuclear factor kappa B (NFκB). Various studies have shown that several SASP cytokines, which are involved in the regulation of bone turnover, are elevated during the aging process. IL-6 is an important example because it increases steadily with aging. Moreover, IL-6 is a potent promoter of osteoclast differentiation and activation, thus supporting bone resorption (Jilka et al., 1992). TNF-α stimulates bone resorption and inhibits new bone formation (Nanes, 2003). Accordingly, we found a higher expression of both IL-6 and TNF-α in osteoblasts enclosing the bone trabeculae of *Runx2CreSod2^fl/fl^* mice as well as in isolated mesenchymal progenitor cells. These findings are strongly compatible with the impact of inflammatory cytokines on the development of osteoporosis (Pietschmann et al., 2016).

The redox transcription factors forkhead box, class O (FoxO) family proteins counteract ROS production by upregulating antioxidant enzymes, including catalase and SOD2, maintaining bone cell function and preserving skeletal homeostasis (Klotz et al., 2015; Teixeira et al., 2010). In aging mice, FoxO-targeted gene expression increases in bone, accompanied by an elevation in oxidative stress marker expression (Almeida et al., 2007). In agreement with this, we detected increased FoxO1 expression, an early molecular regulator of osteoblast differentiation (Teixeira et al., 2010), particularly in osteoblasts lining the bone trabeculae near the growth plate of *Runx2CreSod2^fl/fl^* mice. This implies that FoxO levels enhanced in response to excessive ROS, possibly not only to maintain the osteoblast phenotype, but also to prevent subsequent senescence (Rached et al., 2010).

In conclusion, we demonstrated that mice with an osteoblast lineage-specific *Sod2* deficiency display a low bone mass phenotype due to impaired osteoblast proliferation and differentiation, osteoblast senescence, accumulation of adipocytes in the bone marrow and increased osteoclast activity. This suggests that mitochondrial redox balance in osteoblast lineage cells is indispensable for skeletal homeostasis, and could be an attractive target for therapeutic intervention of age-related bone loss. As osteoblast senescence has not been observed in ovariectomy-induced mice (Farr et al., 2019), our model might open up new possibilities to study this important cellular mechanism of aging. In that regard, application of antioxidative or senolytic drugs might be a promising strategy to reduce the number of senescent and thus dysfunctional cells in osteoporotic bone, which might prevent aggravation of the disease and improve fracture healing. In fact, a senolytic co-treatment using dasatinib and the antioxidant quercetin, was previously described to restore the osteogenic capacity of senescent bone marrow mesenchymal stem cells as demonstrated in enhanced bone repair in aged mice (Zhou et al., 2021). Taken together, the *Runx2CreSod2^fl/fl^* mice represent a suitable model to further investigate bone repair, novel treatment strategies and underlying pathomechanisms in the context of age-related osteoporosis.

## MATERIALS AND METHODS

### Animals

*Sod2^fl/fl^* (C57BL/6 background) and *Runx2Cre* transgenic animals were described previously (Rauch et al., 2010; Strassburger et al., 2005; Treiber et al., 2011). *Runx2Cre* were initially generated on an FVB/N background and backcrossed for at least 10 generations to C57BL/6. Genotyping was performed using the primers 5′-CCAGGAAGACTGCCAGAAGG-3′, 5′-TGGCTTGCAGGTACAGGA G-3′ and 5′-GGAGCTGCCGAGTCAATAAC-3′ detecting a 780 bp wild-type sequence and a 600 bp transgenic *Runx2Cre* sequence. Using the primers 5′-GAGGGGCATCTAGTGGAGAAG-3′ and 5′-AGGAAAGTCACCTCCACACACAG-3′, an 800 bp wild-type allele and a 1081 bp *Sod2^fl/fl^* allele were detected. All mice were housed at two to five per cage under a 12 h/12 h dark/light cycle, and food and water were supplied *ad libitum*. *Runx2CreSod2^fl/fl^* mice were mated with *Sod2^fl/fl^* mice. Organ removal was performed from *Runx2CreSod2^fl/fl^* mice and the littermate control mice. Organ removal was approved by the local ethics committee (Regierungspräsidium Tübingen, o.135-4) and was performed in accordance with the national and international regulations for the care and use of laboratory animals. 12- and 52-week-old female and male mice were used in the study.

### μCT

μCT analysis was performed as described previously (Kemmler et al., 2015). To investigate the bone phenotype of mice, right femurs were fixed in 4% formalin and lumbar vertebrae (L3) were dehydrated in 80% ethanol. Femurs and lumbar vertebrae were scanned using a μCT device (Skyscan 1172, Kontich, Belgium) set at a resolution of 8 μm, a maximum voltage of 50 kV and a power of 200 mA. Reconstruction and analysis were performed using software (NRecon, Data viewer and CT analyzer) from Skycan. Two defined hydroxyapatite (HA) phantoms (250 and 750 mg/cm^3^), scanned together with the bones in each scan, were used to determine BMD. According to the guidelines of the American Society for Bone and Mineral Research (ASBMR), two global thresholds (394 and 642 mg HA/cm^3^) were used to distinguish between mineralized and non-mineralized tissue, respectively. The regions of interest (ROI) were defined as the area between 240 μm and 960 μm from the proximal end of the growth plate of the femur and the entire trabecular area of the vertebral body for the analysis of trabecular bones, and the region from the border of the trochanter to the following 2000 μm for the assessment of the cortical bone. TMD, BV/TV, Tb.N, Tb.Sp, Tb.Th and Ct.Th were selected as the common parameters for bone evaluation (Bouxsein et al., 2010; Kovtun et al., 2017).

### Histomorphometric analysis

Undecalcified sections were stained for the analysis of tissue composition. Methyl methacrylate-embedded sections were immersed in 2-methoxyethyl acetate and a decreasing ethanol series. Plump cuboidal blue cells located on the surface of bone were identified as osteoblasts by Toluidine Blue staining. Cells on the bone surface that were stained red with two or more nuclei were considered to be osteoclasts by tartrate-resistant acid phosphatase (TRAP) staining. Adipocyte number was determined after staining with Hematoxylin and Eosin. Osteoblast number per bone perimeter (N.Ob/B.Pm, mm^−1^), osteoblast surface per bone surface (Ob.S/BS, %), osteoclast number per bone perimeter (N.Oc/B.Pm, mm^−1^) and osteoclast surface per bone surface (Oc.S/BS, %) were measured using Osteomeasure software (Osteometrics, Atlanta, GA, USA). The area at a distance of 200 μm from the growth plate was determined as the region of interest under the light microscope at a 20-fold magnification.

### Immunohistochemistry

Paraffin-embedded sections were prepared as previously described (Haffner-Luntzer et al., 2016). To perform antigen retrieval, slides with sections were immersed in 10 mM citrate buffer (pH 6.0) in a 95°C water bath for 20 min. The sections were incubated with primary antibodies (rabbit polyclonal anti-mouse p16^INK4a^ antibody, 1:1000, ab189034, Abcam, Cambridge, UK; rabbit monoclonal anti-mouse FoxO1 antibody, 1:1000, 2880, Cell Signaling, Danvers, MA, USA; rabbit polyclonal anti-mouse TNF-α antibody, 1:100, ab6671, Abcam; rabbit polyclonal anti-mouse IL-6 antibody, 1:250, bs-0782R, Bioss, Woburn, MA, USA), and with the secondary goat anti-rabbit antibody, followed by incubation with avidin-biotin complex and NovaRed substrate (Vector, Peterborough, UK), or with secondary goat anti-rabbit Alexa Fluor 594 (Thermo Fisher Scientific). Control sections were treated in parallel and incubated with the isotype control antibody (011-000-003, Jackson Immunoresearch, Ely, UK). For nuclei staining, Hematoxylin (Waldeck, Münster, Germany) was used. Before analyzing sections microscopically, the slides were mounted with Vitro-Clud (Langenbrinck, Emmendingen, Germany) or Fluoromount (Sigma Aldrich, Taufkirchen, Germany).

### Cryosectioning of undecalcified bone

Immediately after isolation, mice tibiae were embedded in optimal cutting temperature (OCT) compound (Sakura Finetek, Germany) and frozen in liquid nitrogen. Bones were cut and collected as 10 μm-thick sections on adhesive cryofilm (Section-lab Co, Hiroshima, Japan). The cryosections were used for the SA-β-Gal activity assay.

### Cell isolation and culture

Osteoblasts were generated from long bones of mice as described previously (Rapp et al., 2018). Following digestion with 300 U/ml collagenase type IV (Sigma Aldrich) in alpha Minimum Essential Medium (α-MEM; Biochrom, Berlin, Germany) for 2 h with shaking at 37°C and under 5% CO_2_ in an incubator, the bones were placed in six-well plates in α-MEM supplemented with 10% fetal bovine serum superior (Biochrom), 1% penicillin/streptomycin, 1% L-glutamine and 0.5% amphotericin B (Thermo Fisher Scientific) in a 37°C and 5% CO_2_ incubator. Mesenchymal progenitor cells (MSCs) were isolated from the long bones of mice as described previously (Rapp et al., 2018). Bone marrow cells were seeded at a density of 5.5×10^7^ cells/cm^2^ in expansion medium (MesenCult™ Expansion Kit, Mouse, Stemcell Technologies, Vancouver, Canada). According to the manufacturer's instructions, the MSCs were cultured with additional MesenPure™ at 37°C and under an atmosphere of 6.0% O_2_ and 8.5% CO_2_. Osteoblasts and MSCs in passages 3-5 were used for further experiments.

### DHE staining

Cells were seeded into 24-well plates at a density of 20,000 cells/well and incubated for 24 h. Following rinsing with phosphate-buffered saline (PBS), the cells were incubated with DHE (Sigma Aldrich) diluted in PBS (10 μM) for 30 min at 37°C under 8.5% CO_2_. The samples were washed with PBS, visualized and fluorescence images captured using a microscope with Ex/Em 488/574-595 nm (red) and Ex/Em 352/461 nm (blue) (Nijmeh et al., 2010). DHE staining was quantified in cultured osteoblasts by using ImageJ software (NIH, Bethesda, MD, USA, version 2.0.0) and calculating the CTCF.

### TUNEL assay

The terminal deoxynucleotidyl transferase dUTP nick end labeling (TUNEL) assay was performed as described in the manufacturer's protocol (Biozol, Eching, Germany). Rehydrated paraffin-embedded sections were washed with PBS and immersed with 20 mg/ml proteinase K for 30 min at 37°C. To perform the TUNEL assay, sections were permeabilized with TUNEL reaction solution for 2 h in a humid chamber at 37°C in the dark.

### Gene expression analysis

RNA was harvested using RLT buffer (Qiagen, Hilden, Germany) containing 1% β-mercaptoethanol (Sigma Aldrich) from mesenchymal progenitor cells and osteoblasts after cultivation in proliferation medium for 3 d (d0) followed by cultivation in osteogenic differentiation medium [Dulbecco's modified Eagle medium (DMEM)/F12 (Gibco-Thermo Fisher Scientific) supplemented with 10% fetal calf serum (Merck, Darmstadt, Germany), 0.1 μM dexamethasone, 10 mM β-glycerophosphate disodium, 0.2 mM ascorbate-2-phosphate] for an additional 10 days and 14 days, respectively.

RNA isolation was performed according to the instructions of RNeasy Mini Kit (Qiagen). Total RNA was diluted in RNase-free water, and the RNA concentration was determined by spectrophotometry. A total of 1 μg of total RNA in RNase-free water was used to generate cDNA in a total volume of 20 µl using Omniscript Reverse Transcriptase (Qiagen). qRT-PCR was performed using the SensiFast SYBR Hi-ROX One Step Kit according to the manufacturer's protocol (Stratagene, Amsterdam, Netherlands) and analyzed by QuantStudio 3 (Thermo Fisher Scientific). Primer pairs for the amplification of *Alpl* (5′-GCTGATCATTCCCACGTTTT-3′, 5′-GAGCCAGACCAAAGATGGAG-3′), *Runx2* (5′-CCACCACTCACTACCACACG-3′, 5′-CACTCTGGCTTTGGGAAGAG-3′), *Pparg* (5′-CGTGAAGCCCATCGAGGACAT-3′, 5′-GGGTGGTTCAGCTTGAGCTGCAG-3′), *Rankl* (5′-ATCATGAAACATGGGGAAGC-3′, 5′-CTTGGGATTTTGATGCTGGT-3′), *Opg* (5′-CTGCCTGGGAAGAAGATCAG-3′, 5′-GCTCGATTTGCAGGTCTTTG-3′), *p53* (5′-GGAAATTGTATCCCGAGTATCTG-3′, 5′-GTCTTCCAGTGTGATGATGGAAA-3′), *p21* (5′-CCTCCCAAGATAGCCGAGTT-3′, 5′-AGACGACACAGGTGAGGAAG-3′), *p16^INK4a^* (5′-CTTCCTGGACACGCTGGT-3′, 5′-GTCTTGATGTCCCCGCTCT-3′), *Sod2* (5′-AACTCAGGTCGCTCTTCAGC-3′, 5′-GGTTCTCACCCACCACCCTTAGG-3′), *Cebpa* (5′-CGTCTGCCTCCCAGAGGACCAATTA-3′, 5′-CACCCTTGGACAACTAGGGGAGAGG-3′), *aP2* (5′-GTGACAAGCTGGTGGTGGAAT-3′, 5′-CATCCAGGCCTCTTCCTTTGG-3′), *Plin1* (5′-TACCTAGCTGCTTTCTCGGTG-3′, 5′-CACAGGCAGCTGAACTC-3′), *Tnfa* (5′-GGCCACCACGCTCTTCTGTCTACT-3′, 5′-TGATCTGAGTGTGAGGGTCTGGGC-3′) and *Il6* (5′-TCCTTCCTACCCCAATTTCC-3′, 5′-GCCACTCCTTCTGTGACTCC-3′) were used. The mRNA expression was calculated by the term PCR efficiency^−ΔCt (gene of interest)^. The PCR efficiencies from the different primer pairs were calculated using the software LinRegPCR (Ramakers et al., 2003). Beta-2-microglobulin (*B2m*) was selected as the housekeeping gene.

### Western blot analysis

10 µg of protein lysate from osteoblasts was separated using SDS-PAGE and transferred to a nitrocellulose membrane (BioRad, Hercules, CA, USA). The membranes were incubated with the rabbit monoclonal anti-mouse FoxO1 antibody (Cell Signaling) and the rabbit monoclonal anti-mouse GAPDH antibody (Cell Signaling) overnight at 4°C. Protein bands were visualized as described previously (Liedert et al., 2011).

### BrdU assay and cytological staining

The proliferation assay was performed using the BrdU Cell Proliferation Assay Kit (Cell Signaling) according to the manufacturer's instructions. Osteoblasts were seeded into 96-well plates at 2000 cells/well and were incubated with BrdU reagent for 24 h.

AP staining was performed to detect AP activity using an alkaline phosphatase kit (Sigma Aldrich). Following incubation with osteogenic differentiation medium for 14 days, cells were fixed with citrate-acetone-formaldehyde fixing solution for 2 min. The AP staining solution was prepared immediately prior to staining. For the staining process, the cells were incubated with the AP staining solution, and the plates were kept in the dark for 15 min. The cells were washed with distilled water and images were captured using a digital camera.

Alizarin Red and Von Kossa staining were used to analyze matrix mineralization of differentiated osteoblasts. Cells were fixed with 4% formalin solution for 15 min followed by incubation with 0.1% Alizarin Red staining solution (Sigma Aldrich) for 30 min. For Von Kossa staining, the cells were incubated with 5% silver nitrate solution for 60 min at room temperature (in the dark). Following rinsing with distilled water, the cells were incubated with 1% pyrogallol solution for 5 min followed by incubation with 5% sodium thiosulfate solution for 5 min. Adipocytes were detected using 0.2% Oil Red O staining solution after cultivation for 3 d in adipogenic differentiation medium (α-MEM supplemented with 1 μM dexamethasone, 0.1 mM indomethacin, 0.5 mM 3-isobutyl-1-methylxanthine and 10 μg/ml insulin) and successive cultivation in proliferation medium for 7 days.

### SA-β-Gal activity assay

According to the manufacturer's protocol (Sigma Aldrich), osteoblasts were seeded into six-well plates at a density of 40,000 cells/well and fixed with fixation buffer for 7 min. The cells were immersed in PBS and incubated with the staining mixture solution at 37°C without CO_2_ overnight. The senescent cell ratio was assessed as the ratio of the total number of stained (blue) cells to the total number of cells counted (≥1000) in one well.

Bone cryosections were prepared to perform SA-β-Gal staining. Following washing with PBS, the bone cryosections were fixed with fixation buffer for 7 min and incubated with the staining mixture solution at 37°C overnight. The slides were dried and mounted with Fluoromount™ aqueous mounting medium before capturing images using a microscope.

### Data analysis

Data are presented as mean±s.d. IBM SPSS statistics version 24 software and GraphPad Prism version 6.07 software were used to analyze data. Following evaluation for normality by Shapiro–Wilk test, data were analyzed by two-tailed unpaired *t*-test, one-way ANOVA with Fisher's LSD post hoc test and two-way ANOVA with Sidak multiple comparison test. *P*<0.05 was considered statistically significant.

## Supplementary Material

Supplementary information
