# Supplementary material for: Osteoblast lineage Sod2 deficiency leads to an osteoporosis-like phenotype in mice
Source: Dis Model Mech. 2022 May 13;15(5):dmm049392. doi: 10.1242/dmm.049392 (PMC9118037; doi:10.1242/dmm.049392)
Supplement: Supplementary information [file dmm-15-049392-s1.pdf]

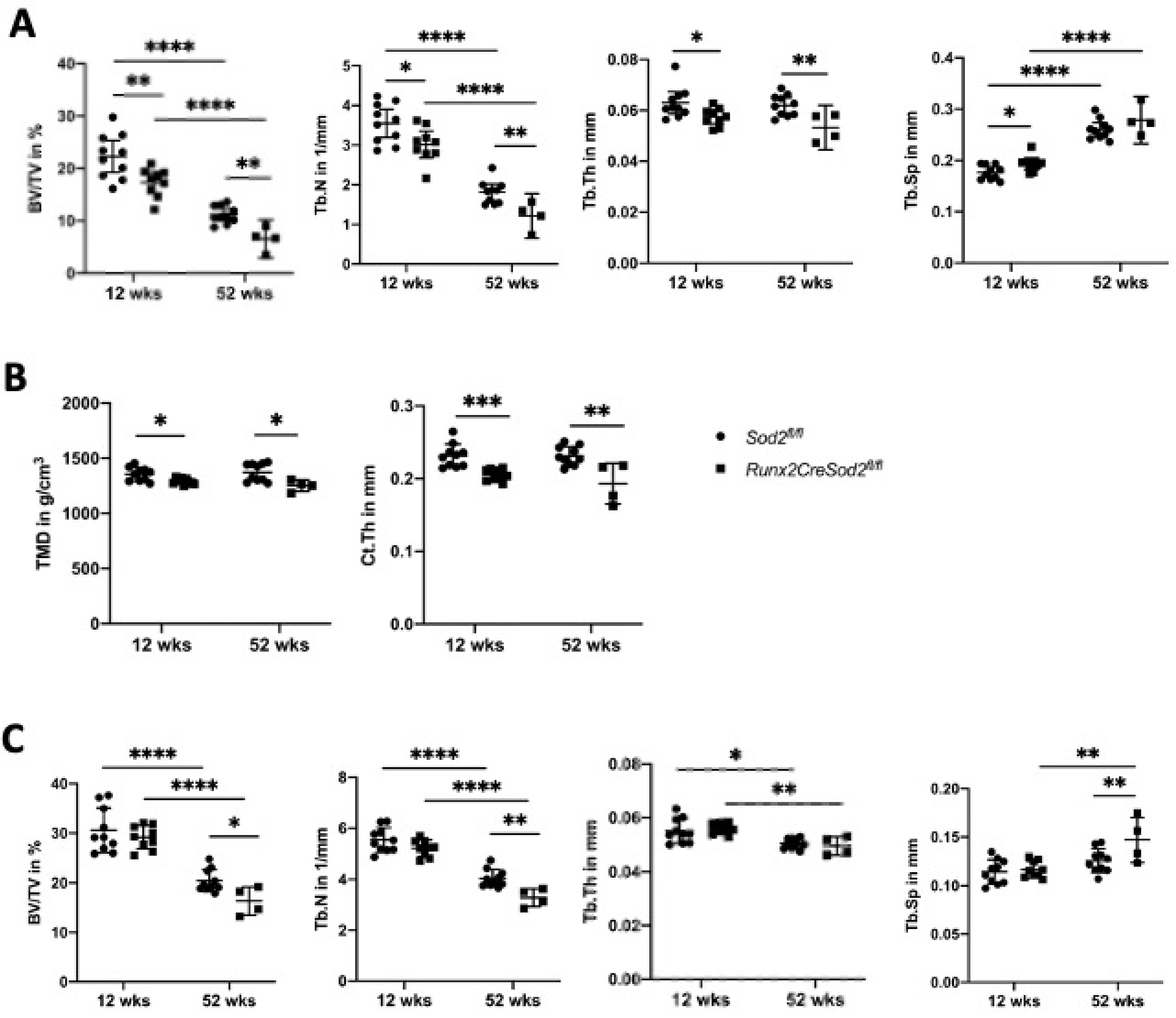

A
BV/TV in %
Tb.N in 1/mm
Tb.Th in mm
Tb.Sp in mm
12 wks
52 wks
B
TMD in g/cm3
Ct.Th in mm
Sod2fl/fl
Runx2CreSod2fl/fl
C

Fig. S1. Bone mass loss in osteoblast lineage-specific *Sod2*-deficient male mice aged 12 and 52 weeks.

(A) µCT-based quantification of the trabecular bone volume per tissue volume ratio (BV/TV), trabecular number (Tb.N), trabecular thickness (Tb.Th) and trabecular separation (Tb.Sp) in the distal femora of male *Runx2CreSod2fl/fl* mice (■) compared to male *Sod2fl/fl* mice (●). (B) µCT-based quantification of the tissue mineral density (TMD) and cortical thickness (C.Th) of the femora of male *Runx2CreSod2*$^{fl/fl}$ mice (■) compared to male *Sod2*$^{fl/fl}$ mice (●). (C) Histomorphometric quantification of BV/TV, Tb.N, Tb.Th, and Tb.Sp in the vertebral bodies of male *Runx2CreSod2*$^{fl/fl}$ mice (■) compared to male *Sod2*$^{fl/fl}$ mice (●) determined by µCT. Bars represent mean ±SD (n=4-10/group). Asterisks indicate statistically significant differences between the groups (* <0.05, ** <0.01, *** <0.001, **** <0.0001).

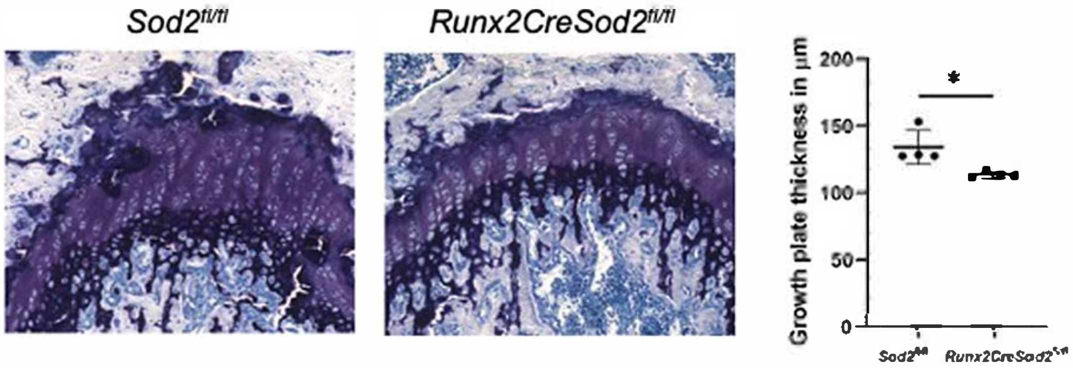

Fig. S2. Decreased growth plate thickness in female mice with osteoblast lineage-specific *Sod2* deficiency. Representative images of femur cross-sections stained with toluidine blue showing the growth plate from female *Sod2*$^{fl/fl}$ mice and *Runx2CreSod2*$^{fl/fl}$ mice. Quantification of growth plate thickness in femur cross-sections from *Sod2*$^{fl/fl}$ mice (●) and *Runx2CreSod2*$^{fl/fl}$ mice (■) Bars represent mean ±SD (n=4/group). Asterisks indicate statistically significant differences between the groups (* <0.05)

Disease Models & Mechanisms • Supplementary information
